# Atrial fibrillation recurrence after rhythm control: gaps in outpatient risk factor management in a single-centre retrospective cohort study

**DOI:** 10.1186/s12872-026-05851-4

**Published:** 2026-04-23

**Authors:** D. Murphy, K. Strong, J. Graby, J.C.L. Rodrigues, D. Thompson, A. Khavandi

**Affiliations:** 1 Cardiology Department, Royal United Hospitals Bath NHS Foundation Trust, Bath, UK; 2 Department of Health, University of Bath, Bath, UK; 3 Radiology Department, Royal United Hospitals Bath NHS Foundation Trust, Bath, UK

**Keywords:** Atrial fibrillation, Cardioversion, Catheter ablation, Risk factors, Disease recurrence, Lifestyle

## Abstract

**Background:**

Guidelines recommend systematic identification and management of modifiable risk factors (RFs) in atrial fibrillation (AF) to reduce recurrence and improve outcomes. The effectiveness of routine, single-visit outpatient interventions remain unclear. This study evaluates the screening, prevalence, control, and initial management of modifiable RFs in patients referred for direct current cardioversion (DCCV) or catheter ablation (CA), and their association with AF recurrence.

**Methods:**

In this single-centre retrospective cohort study, 325 consecutive AF patients referred for DCCV or CA over two years were analysed. Electronic records were reviewed for RF screening (hypertension, elevated body mass index, glycaemic control, sleep-disordered breathing, alcohol misuse), RF control status, and documentation of RF management at their index clinic visit. AF recurrence was ascertained from follow-up documentation. AF-free survival was evaluated using Kaplan-Meier analysis and log-rank testing.

**Results:**

Risk-factor screening was commonly undertaken, with 70% having at least four of five modifiable risk factors assessed. Overall, 89% had ≥ 1 risk factor identified, and 71% had at least one uncontrolled risk factor at the index outpatient visit. Among those with ≥ 1 uncontrolled risk factor, only 14% had documentation that all uncontrolled risk factors were actively managed at that encounter, while 70% had no documented active management. During a median follow-up of 23 months, AF recurrence occurred in 61%. AF-free survival did not differ according to whether uncontrolled risk factors were fully, partially, or not managed at the index visit (log-rank *p* = 0.95). These findings were unchanged after adjustment for age, sex, procedure type, and AF phenotype.

**Conclusions:**

These findings highlight both the limited impact of isolated clinic encounters and the low rate of active risk-factor management in routine practice, supporting the need for more systematic and sustained approaches to risk-factor modification.

## Introduction

 Atrial fibrillation (AF) is the most common sustained cardiac arrhythmia, affecting approximately 1.5 million people in the UK [1]. Patients with AF frequently experience impaired health-related quality of life (HRQOL) and reduced functional capacity [2]. AF is a leading cause of tachycardia-induced cardiomyopathy and heart failure [3], accounts for approximately one-third of ischaemic strokes [4], and independently confers a 1.5-1.9-fold increase in mortality [5]. It is associated with recurrent hospitalisations and substantial healthcare utilisation, with direct National Health Service (NHS) costs projected to rise to 1.35–4.27% of total expenditure over the next two decades [6]. Strategies that reduce AF-related morbidity and hospitalisation therefore have significant clinical and economic implications.

AF is a progressive condition, often beginning with brief, self-terminating episodes (paroxysmal AF [pAF]) that may evolve into persistent forms over time [7]. Key modifiable risk factors including elevated body mass index (BMI), hypertension (HTN), impaired glycaemic control, and sleep-disordered breathing (SDB), contribute not only to AF onset but also to disease progression. Increasing evidence suggests that these cardiometabolic factors promote structural and electrical remodelling of the atria, resulting in an atrial cardiomyopathy that sustains AF [8]. When unaddressed, this substrate reduces the likelihood of durable success following rhythm-control interventions such as catheter ablation (CA) or direct current cardioversion (DCCV). Thus, rhythm restoration in isolation, without addressing underlying modifiable risk factors, may yield suboptimal long-term outcomes.

Several studies have demonstrated associations between individual risk factors and AF recurrence. Uncontrolled hypertension is associated with higher rates of AF recurrence following CA and a greater need for extensive ablative strategies [9]. SDB is highly prevalent in patients with AF [10] and increases the risk of recurrence following both DCCV and CA, whereas effective treatment reduces ablation failure rates [11, 12]. Hyperglycaemia promotes atrial fibrosis and has been linked to AF incidence and recurrence, with elevated glycated haemoglobin (HbA1c) levels associated with higher recurrence rates and repeat procedures [13–15]. Obesity demonstrates a dose-dependent relationship with AF incidence and recurrence, and weight reduction has been shown to improve symptom burden and rhythm outcomes [16–19]. 

Accordingly, international guidelines recommend routine identification and management of modifiable risk factors as a core component of AF care [20]. However, in routine clinical practice, implementation often consists of brief, single-visit counselling or medication alterations. Both patients and clinicians recognise that current healthcare structures are frequently insufficient to deliver sustained risk-factor modification [21, 22]. 

Despite strong evidence linking risk factors to AF progression and recurrence, it remains unclear how consistently routine outpatient cardiology services operationalise guideline-recommended risk factor management, and whether opportunistic, single-visit interventions translate into improved rhythm outcomes.

This study aimed to evaluate, in patients referred for DCCV or CA: (1) the extent of screening for common AF risk factors; (2) the prevalence and control of these risk factors at clinical review; and (3) whether active management was initiated when abnormalities were identified. We further examined the association between risk factor burden, outpatient management, and AF recurrence following intervention. We hypothesised that risk factors would be frequently identified but incompletely controlled, and that documentation of management at a single clinic visit would not be associated with reduced AF recurrence.

## Methods

### Ethics approval and consent to participate

This study involved a retrospective review of clinical documentation only. According to the UK Health Research Authority (HRA) decision tool, the project was classified as a service evaluation and therefore did not require review by a Research Ethics Committee or formal ethical approval [23]. The study was registered as a service evaluation with the Trust’s audit department, which confirmed that formal written informed consent from participants was not required. The study was conducted in accordance with the principles of the Declaration of Helsinki. Patients and members of the public were not involved in the design, conduct, reporting, or dissemination of this work.

### Study design and setting

This was a single centre retrospective observational cohort study of consecutive AF patients referred for a DCCV and/or CA over a two-year period (2021/2022). Our institution is a district general hospital (DGH) in the South-West of England with a bed capacity of approximately 750. DCCV are performed on site, while all CA procedures are carried out at the nearby tertiary referral centre.

### Data collection

A retrospective database was created using all referrals for DCCV and CA submitted by the cardiology service between January 2021 and 2022.

We manually reviewed the electronic patient records, including general practitioner (GP) referral letters to the cardiology service, documentation from the index cardiology clinic consultation, and any relevant clinical correspondence from secondary care. The index clinic visit was defined as the cardiology outpatient consultation during which the decision for rhythm-control intervention was documented.

Information was collected on the following risk factors:

- HTN
- BMI
- Diabetes mellitus (DM) or impaired glucose tolerance(IGT)
- SDB
- Alcohol use

Data was extracted into a structured spreadsheet using binary fields to standardise the recording of risk factor screening and control status.

### Risk factor screening

A risk factor was considered to have been screened for if there was documentation in the clinical records, either within the GP referral, the index cardiology clinic consultation, or associated secondary care correspondence, that indicated the risk factor had been acknowledged, measured, or discussed. Screening was assessed for each individual risk factor. Specific definitions for each were as follows:

- HTN: Reference to hypertension diagnosis or anti-hypertensive treatment.
- BMI: Documentation of patient BMI.
- Diabetes mellitus or IGT: Mention of a diagnosis, treatment, or recorded HbA1c within three months of the clinical appointment.
- SDB: Any reference to symptoms suggestive of SDB, prior diagnosis (e.g. obstructive sleep apnoea), or referral to sleep study.
- Alcohol use: Any documented comment on alcohol consumption.

If no such information was available for a given risk factor, it was considered not screened for. 

### Risk factor control at the time of appointment

For each modifiable risk factor, control status was determined using the most recent documented measurement or clinical note available at the time of the index cardiology clinic visit. A risk factor was considered uncontrolled if it met the following thresholds:

- HTN: In patients with an established diagnosis of HTN: systolic blood pressure ≥140 mmHg and/or diastolic blood pressure ≥90 mmHg [24].
- BMI: BMI ≥ 27 kg/m². The selected threshold reflects a clinically relevant cut-off used in prior AF risk factor modification studies, where achieving a BMI <27 kg/m² was associated with improved AF outcomes [17].
- Glycaemic control: For glycaemic status, we defined ‘dysglycaemia’ as patients with an established diagnosis of DM: HbA1c ≥53 mmol/mol. In patients without a diagnosis of DM or IGT: HbA1c 42-47 mmol/mol (indicative of IGT) or HbA1c >47 mmol/mol (diagnostic of DM) within three months of the clinic appointment, i.e. newly identified IGT/DM [25].
- SDB: Patients with a diagnosis of SDB who were not referred for treatment, remained symptomatic without further evaluation were classified as having uncontrolled SDB. Additionally, patients with a new diagnosis at the time of the clinic visit were also considered to have uncontrolled SDB.
- Alcohol use: Consumption exceeding UK recommended limits, or documentation of harmful or heavy use [26].

### Assessment of risk factor management

For patients in with ≥1 uncontrolled modifiable risk factor, we assessed whether a documented management plan was initiated at the index consultation. An uncontrolled risk factor was considered actively managed when one of following actions were documented:

- Initiation or adjustment of pharmacological therapy (e.g. anti-hypertensives or glucose-lowering medications).
- Referral to another specialist service (e.g. sleep clinic for SDB, diabetes team, or dietetics department).
- Provision of lifestyle advice or counselling (e.g. on weight reduction, alcohol reduction, or physical activity).
- Scheduling of follow-up specifically to reassess the identified risk factor.

Where no such action was documented, the risk factor was considered unmanaged. Complete risk factor management was defined as all identified uncontrolled risk factors being managed at clinical review. Partially managed was defined as at least one uncontrolled risk factor being actively managed, without all risk factors meeting these criteria. Unmanaged was defined as the absence of any active management for any identified uncontrolled risk factor.

### Assessment of AF recurrence

AF recurrence was assessed based on a repeat presentation to our secondary care service (e.g. the emergency department) with documented AF or through review of follow-up clinical documentation, including cardiology clinic letters and discharge summaries. Recurrence was defined as any documented episode of AF following the index rhythm control procedure (either DCCV or CA), irrespective of symptom status. 

### Statistics

Data are presented as mean and standard deviation (SD) or median and interquartile range (IQR) where appropriate. Continuous parametric data were compared using t-tests, and non-parametric data with the Mann–Whitney U test. Categorical variables were compared using chi-square tests or Fisher’s exact test where appropriate. AF-free survival was assessed using Kaplan-Meier analysis, with differences between groups evaluated using the log-rank test. Cox proportional hazards regression was performed to estimate hazard ratios (HRs) and 95% confidence intervals (CIs). Multivariable Cox regression models were adjusted for age, sex, procedure type (DCCV vs CA), and AF phenotype (paroxysmal vs persistent/long-standing persistent). Interaction between risk-factor burden and management status was assessed by inclusion of a multiplicative interaction term in the Cox model. All analysis was conducted using R version 4.5.0 (2025-04-11) (R Foundation for Statistical Computing, Vienna, Austria). All tests were two-tailed with p<0.05 deemed statistically significant.

## Results

### Study population

**Fig. 1:**
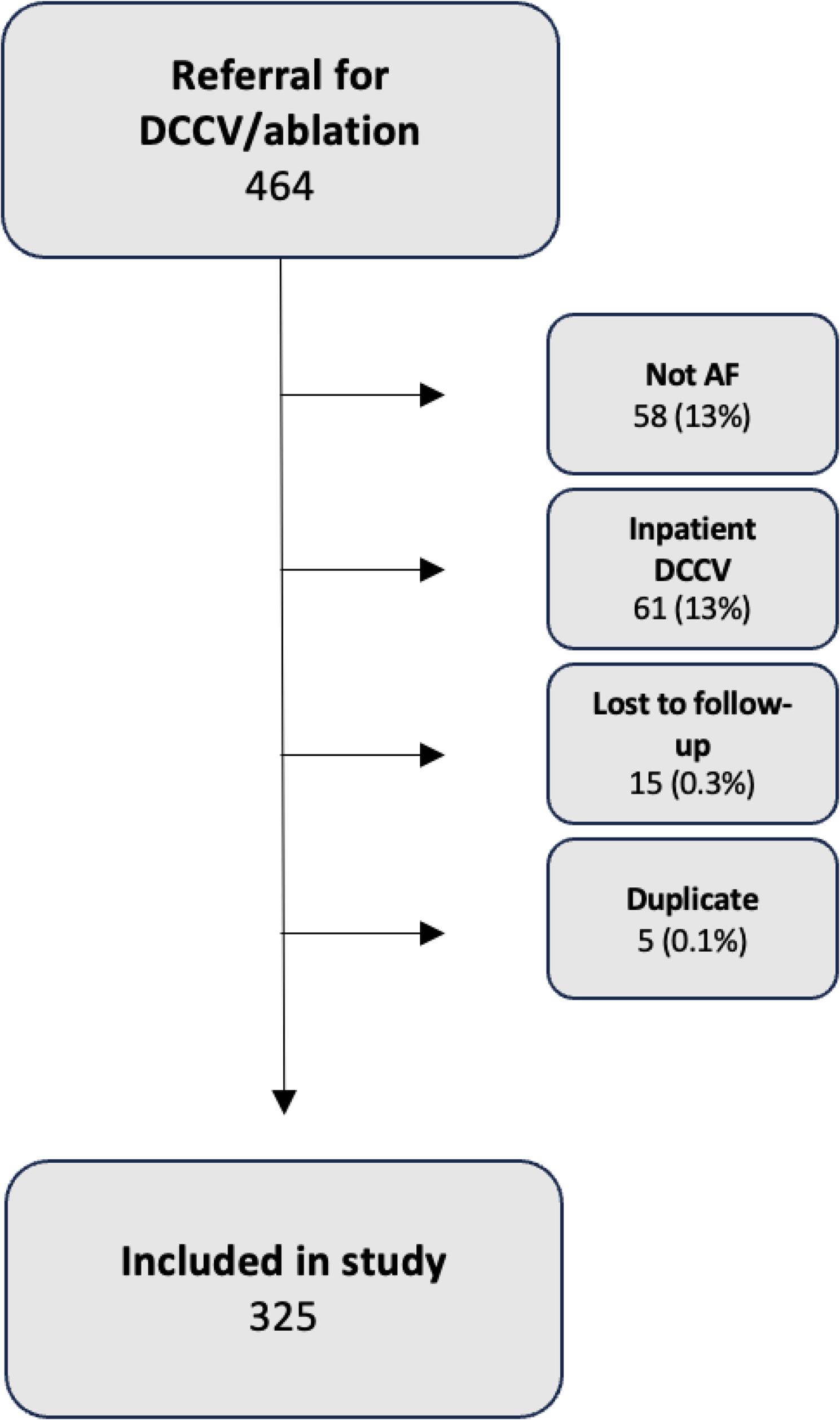
Study flow diagram. DCCV direct current cardioversion, AF atrial fibrillation

A total of 325 patients were included in the study, of whom 68% were male, with a mean age of 62 SD 11 years. The majority, 74% (240/325), were referred for DCCV, while 26% (85/325) were referred for CA. Patient demographics and AF characteristics are summarised in Table 1, and the patient selection process is illustrated in Fig. 1 (flow diagram).

**Table 1:** Baseline characteristics of patients with atrial fibrillation stratified by rhythm-control management strategy

	Total(n=325)	Referred for DCCV (n=240)	Referred for CA (n=85)	*P*-value
Male sex, n (%)	222 (68)	166 (69)	56 (66)	0.6
Mean age in years (SD)	62 (11)	62 (11)	63 (11)	0.5
AF characteristics				
Type of AF• pAF, n (%)• Persistent, n (%)• Long standing persistent, n (%)	74 (23)221 (68)30 (9)	23 (10)189 (78)28 (12)	51 (60)32 (38)2 (2)	< 0.01
Use of anti-arrhythmic medication• Flecainide, n (%)• Amiodarone, n (%)• None, n (%)	32 (10)20 (6)273 (84)	12 (5)15 (6)213 (89)	20 (24)5 (6)60 (71)	0.03
Rate controlling medications• Beta-blockers, n (%)• Calcium channel blockers, n (%)• Digoxin, n (%)• Multiple agents, n (%)• None, n (%)	202 (62)20 (6)8 (2)69 (21)26 (8)	149 (62)12 (5)4 (2)57 (24)18 (8)	53 (62)8 (9)4 (5)12 (14)8 (9)	0.1

Demographics, atrial fibrillation phenotype, and medical therapy are shown for patients referred for direct current cardioversion (DCCV) or catheter ablation (CA). Group comparisons were performed using appropriate statistical tests

*pAF *paroxysmal atrial fibrillation

### Risk factor screening and prevalence

Overall, 10% (32/325) of patients had documented screening for all five modifiable risk factors. The most frequently omitted was an assessment for SDB. Four of the five risk factors were screened in 60% (196/325) of cases.

At least one modifiable risk factor was identified in 89% (288/325). Elevated BMI (≥27 kg/m²) was present in 64% (208/325), hypertension in 46% (149/325), diabetes mellitus in 29% (93/325), alcohol intake exceeding recommended limits in 20% (65/325), and SDB in 11% (35/325) (Fi. 2).

**Fig. 2:**
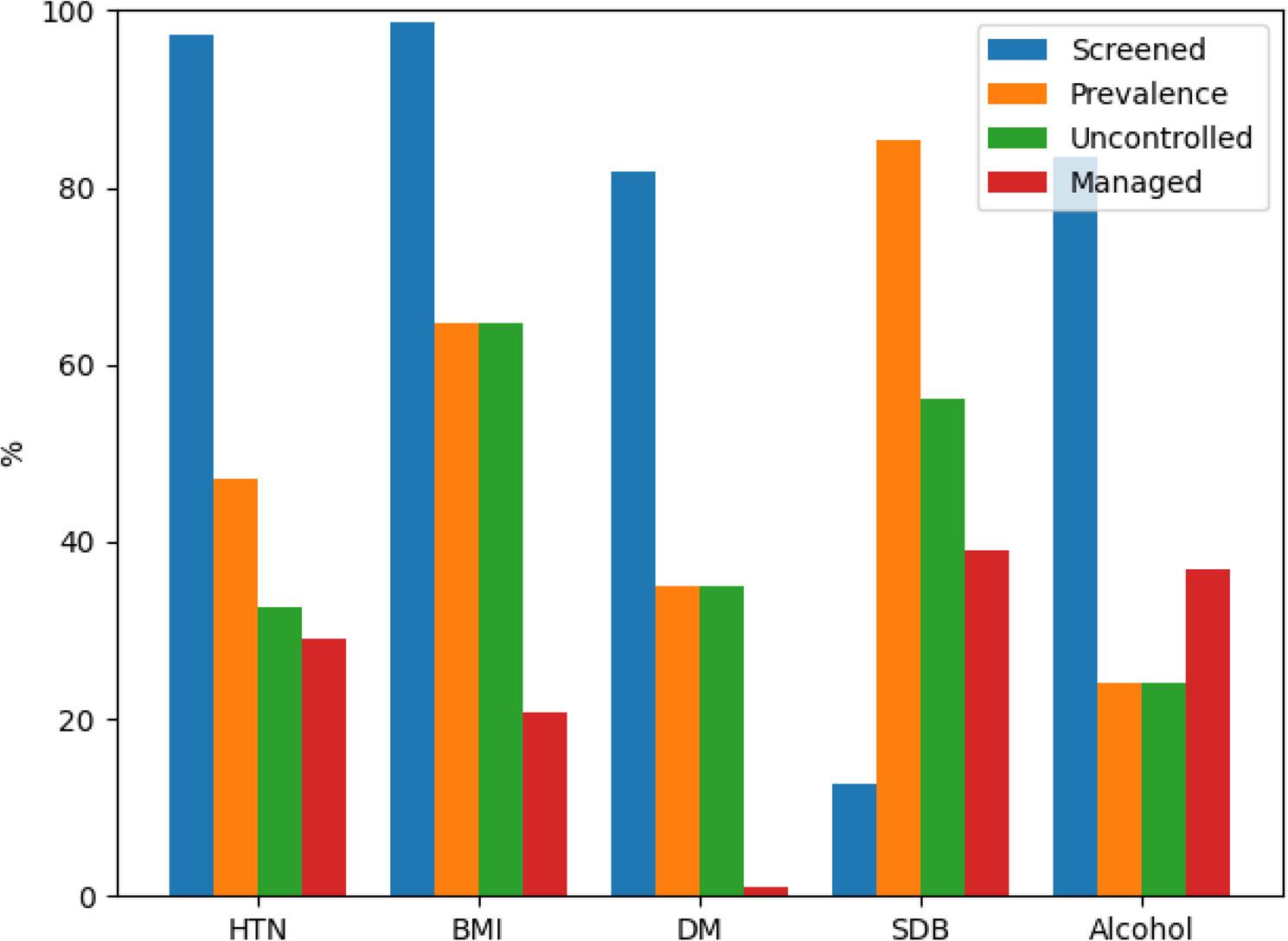
Atrial fibrillation risk factor screening, risk factor prevalence, risk factor control, and risk factor management at the index cardiology clinic visit. The proportion of patients screened for each risk factor; prevalence for each risk factor in the study population; in patients with a risk factor the proportion uncontrolled; and among patients with an uncontrolled risk factor, the proportion actively managed at the time of the index cardiology clinic appointment. HTN=arterial hypertension. SDB=sleep-disordered breathing. BMI=body mass index. HbA1c=glycated haemoglobin

Thirty-seven patients (11%) had no modifiable risk factors. One, two, three, four, and five risk factors were present in 35%, 33%, 16%, 4%, and 1% of patients, respectively.

### Risk factor control and management 

**Fig. 3:**
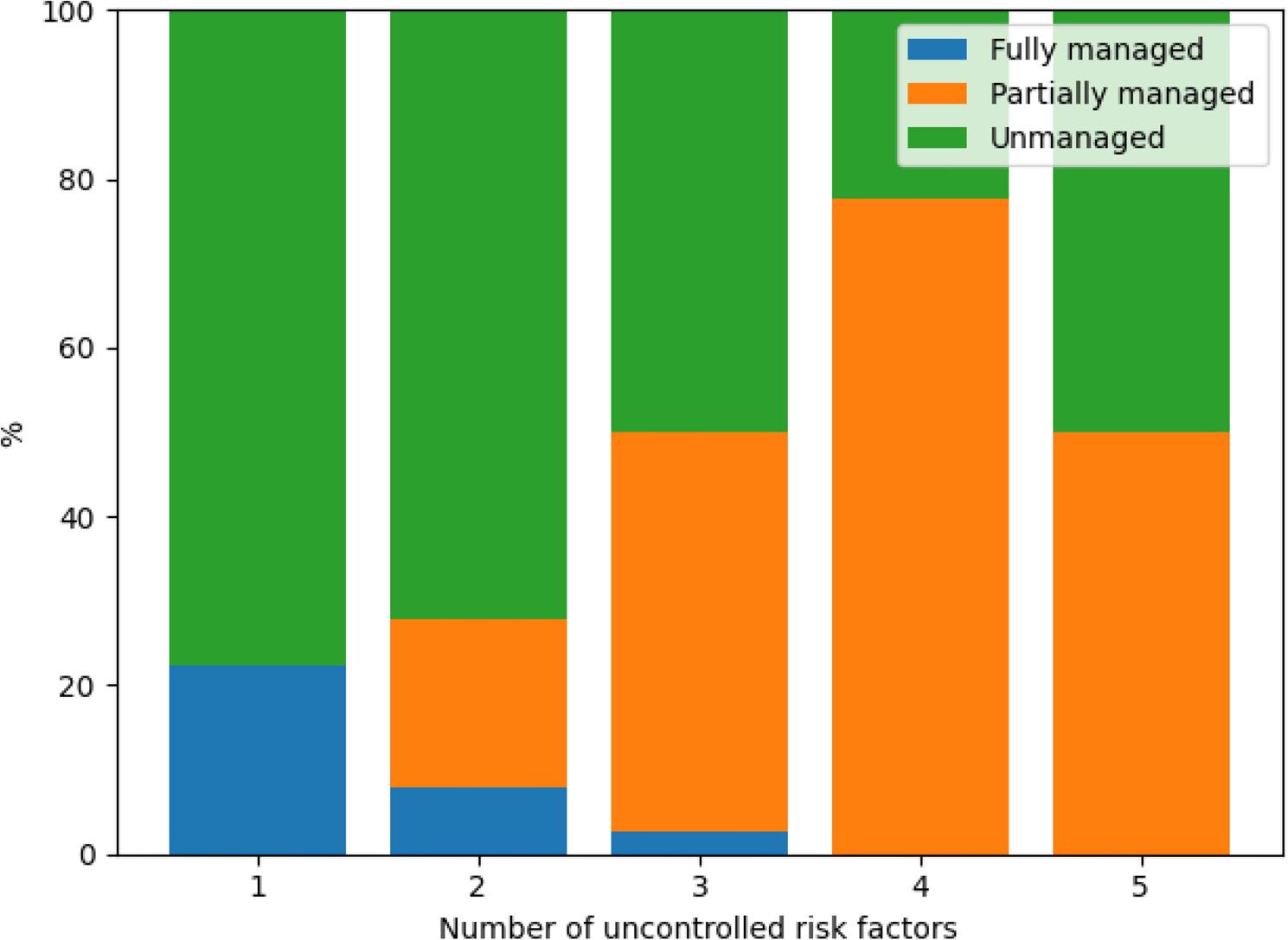
Risk factor control stratified by number of risk factors. Controlled risk factor indicates all identified risk factors were either controlled at clinical review, or if uncontrolled, actively managed. Partially controlled RF indicates at least one risk factor was controlled or managed, but not all. Uncontrolled RF indicates no identified RFs were controlled or actively managed. RF=risk factor

Among patients with at least one identified risk factor, Fig. 2 shows the proportion of risk factors that were uncontrolled at the time of clinical review. Figure 3 provides a breakdown of the management of risk factors that were identified as both present and uncontrolled at clinical review. 

Overall, a substantial proportion of risk factors remained uncontrolled. Patients with multiple risk factors were more likely to have at least one uncontrolled factor, with the proportion of patients achieving complete control decreasing as the number of risk factors increased. Among individual risk factors, HTN and elevated BMI were most frequently uncontrolled, and a significant proportion of these were not actively managed. No documented management plan was recorded for patients with newly identified or uncontrolled diabetes mellitus.

### Follow-up and recurrence

A median follow-up of 23 (IQR 18-33) months from index clinic visit was undertaken. During follow-up, 82% (266/325) underwent a DCCV or CA procedure, of whom 86% received a DCCV and 14% underwent a CA. Documented AF recurrence occurred in 61% of patients, with a median AF-free survival of 9.1 months (95% CI 4.4-13.1).

### Risk factor burden and recurrence

**Fig. 4:**
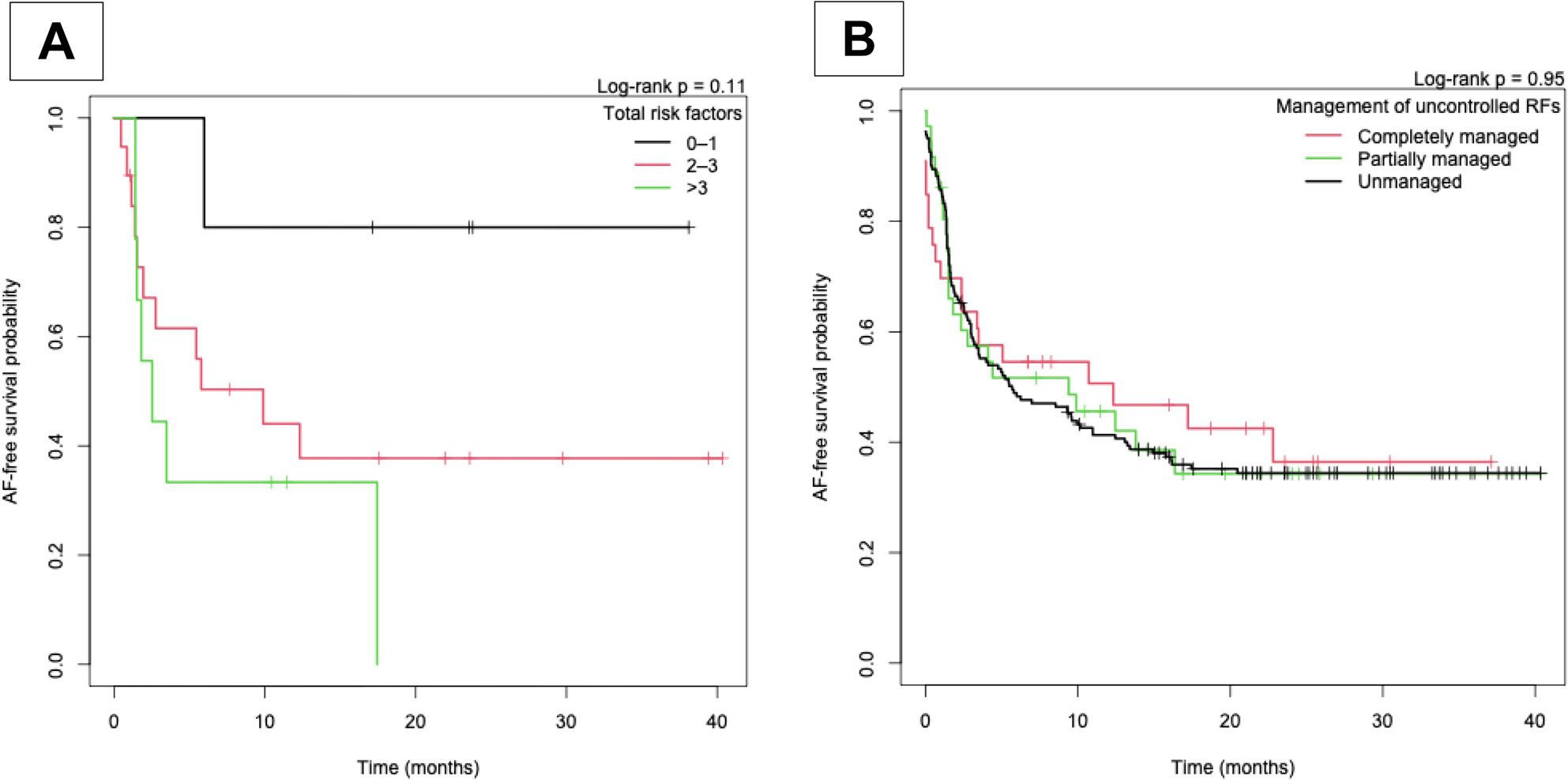
Kaplan Meier curves for AF-free survival. Panel **A** AF-free survival stratified by the number of modifiable risk factors per patient. Panel **B** AF-free survival among patients with ≥1 uncontrolled risk factor, stratified by whether risk factors were fully, partially, or not managed at the index clinic visit. RF=risk factor, AF=atrial fibrillation

Figure 4A demonstrates the Kaplan-Meier curves for AF free survival stratified by the number of risk factors.

AF-free survival decreased numerically with increasing risk factor burden (Fig. 4A). Kaplan-Meier analysis showed no statistically significant difference in recurrence across burden categories (log-rank p=0.11).

In Cox regression, patients with >3 risk factors had a higher estimated risk of recurrence compared with those with 0-1 risk factors (HR 1.81, 95% CI 0.96-3.40), although this did not reach statistical significance. Similarly, each additional uncontrolled risk factor was associated with an estimated increase in recurrence risk (HR 1.46, 95% CI 0.99-2.14), but this association was not statistically significant.

### Outpatient management and recurrence

Among patients with ≥1 uncontrolled risk factor (n=230), 14% were completely managed, 16% partially managed, and 70% unmanaged at the index clinic visit. Kaplan-Meier analysis demonstrated no difference in AF-free survival according to outpatient management status, log-rank p=0.95 (Fig. 4B).

In unadjusted Cox regression, neither partial management (HR 1.07, 95% CI 0.58-1.98; p=0.83) nor absence of management (HR 1.08, 95% CI 0.66-1.76; p=0.76) was associated with recurrence compared with complete management.

In sensitivity analysis restricted to early recurrence (≤6 months), management status likewise remained unassociated with recurrence risk.

These findings were unchanged after adjustment for age, sex, procedure type, and AF phenotype (partially managed vs completely managed: HR 1.05, 95% CI 0.56-1.97; unmanaged vs completely managed: HR 1.01, 95% CI 0.62-1.66).

Finally, outpatient management did not modify the association between uncontrolled risk factor burden and AF recurrence (global interaction p=0.9).

## Discussion

In summary, our key findings are that we found that patients attending their index cardiology appointment were commonly screened for modifiable AF risk factors. These risk factors were highly prevalent and frequently uncontrolled at the time of review. Despite this, documented active management was initiated in only a minority of patients with uncontrolled risk factors. Among patients with ≥1 uncontrolled risk factor, AF recurrence rates were similar regardless of whether management was documented at the index clinic visit, although this reflects documentation at a single timepoint and may not capture the intensity, adherence or longitudinal effectiveness of risk factor modification. Together, these findings suggest that opportunistic, single-visit interventions delivered within routine outpatient care may be insufficient to meaningfully influence long-term rhythm outcomes. They highlight a persistent gap between guideline recommendations and their implementation in everyday practice.

In the context of prior studies such as ARREST-AF, LEGACY, and CARDIO-FIT comprehensive, structured risk-factor management has been shown to significantly reduce AF burden and improve rhythm durability. [17, 27, 28] However, the benefits observed in these studies were achieved through intensive, multidisciplinary programmes rather than standard one-off outpatient consultations. In ARREST-AF improvements in lipid, glycaemic and blood pressure control was seen in the intervention group, but this required three monthly outpatient visits specifically targeting these risk factors. LEGACY demonstrated that sustained weight loss exceeding 10% was associated with significantly improved AF-free survival, however the LEGACY protocol again required structured follow-up every three months with regular review thereafter and for those not achieving the target weight loss meal replacement was introduced. Similarly, CARDIO-FIT demonstrated that improvements in cardiorespiratory fitness were associated with reductions in AF burden. Again, this was facilitated by supervised exercise programmes, initially for 20 minutes three times weekly and increasing to at least 200 minutes of moderate intensity exercise per week. Such outcomes are unlikely to be replicated through brief clinic-based appointments often limited to 30 minutes duration. The contrast between these studies and our cohort likely reflects differences in the intervention intensity, continuity, and patient engagement which are unlikely to have equivalent biological impact. Ultimately these studies demonstrate efficacy under controlled, intensive conditions, our findings reflect the limited impact of opportunistic care in routine practice.

From a clinical perspective, these findings suggest that current outpatient models may be poorly configured to deliver the type of risk factor management endorsed by AF guidelines. If risk factor modification is to influence rhythm outcomes, it may need to be embedded within structured longitudinal pathways, dedicated AF risk-factor clinics, or multidisciplinary services with defined follow-up, behavioural support, and treatment escalation. Routine documentation of advice or treatment initiation at one visit should not be assumed to represent effective risk factor control and meaningful longitudinal intervention.

From a mechanistic perspective our findings do not contradict the established biological relationship between cardiometabolic risk factors and AF progression. Obesity, hypertension, and sleep-disordered breathing promote atrial stretch, inflammation, fibrosis, autonomic dysfunction, and electrical remodelling, thereby sustaining an arrhythmogenic substrate. Emerging evidence from advanced cardiovascular imaging further supports this paradigm, with imaging-derived biomarkers of inflammation and perivascular adipose tissue providing additional insights into the structural and inflammatory substrate underlying AF [29]. Short-term or low-intensity interventions may therefore fail to produce an immediate rhythm benefit, particularly when structural atrial changes are already established. Furthermore, any beneficial effect of risk factor treatment may lag behind the rhythm-control procedure itself, especially if reverse remodelling requires months of sustained physiological improvement. Although increasing uncontrolled risk factor burden was associated with a numerical trend toward higher AF recurrence, this did not reach statistical significance; this may reflect limited power, and the finding should be interpreted as hypothesis-generating rather than evidence of no clinically relevant gradient.

This study benefits from real-world data and a clinically relevant cohort of patients undergoing rhythm-control intervention. However, several limitations merit consideration. First, this was a single centre study, which may limit generalisability, although practices in comparable district general hospitals are likely similar. Second, screening and management were defined by documentation introducing the potential for documentation bias. Additionally, this may not fully reflect the true extent or quality of clinical assessment and intervention, and may underestimate management delivered in practice, particularly informal counselling regarding lifestyle modification. In addition, variability in documentation practices between clinicians may have contributed to heterogeneity in recorded management. Incomplete screening may also have led to underestimation of true risk factor burden, as undocumented risk factors could not be captured in the analysis. AF recurrence was identified from clinical documentation rather than systematic rhythm monitoring, so asymptomatic episodes may have been missed. However, clinically relevant AF recurrence is what typically guides management decisions. Third, we did not assess longitudinal changes in risk-factor parameters and therefore cannot determine whether documented management resulted in sustained physiological improvement. We also lacked data on adherence, so documented management may not reflect treatment uptake or persistence. Risk factor management was heterogeneous, and our study design could not quantify the intensity or quality of individual interventions. Finally, residual confounding remains possible, including from clinical variables not fully captured in the dataset, such as other determinants of atrial substrate and recurrence (e.g. heart failure and left atrial size). In addition, the cohort included both DCCV and catheter ablation patients, differences in efficacy between these rhythm-control strategies may have contributed to outcome heterogeneity. However, this reflects real-world clinical practice and current guidelines do not distinguish risk-factor management according to rhythm-control strategy.

## Conclusion

In summary, AF risk factors were highly prevalent and frequently uncontrolled at the time of cardiology review. Documentation of management at a single outpatient encounter was not associated with improved AF-free survival. These findings should not be interpreted as evidence against the effectiveness of risk factor modification itself, but rather highlight the limitations of opportunistic, single-visit interventions delivered without structured follow-up and may support the need for systematic, longitudinal risk-factor management programmes to translate guideline recommendations into improved clinical outcomes.

## Data Availability

The datasets generated and analysed during the current study are not publicly available but are available from the corresponding author on reasonable request.
